# Higher hypertension prevalence, lower incidence, and aggressive treatment with decreasing mortality, cardiovascular, and cerebrovascular incidence in Taiwan from 2005 to 2010: A 2 population-based cohorts study

**DOI:** 10.1097/MD.0000000000022437

**Published:** 2020-09-25

**Authors:** Chia-Te Liao, Pei-Chih Wu, Jung-Chang Shih, Tain-Junn Cheng, Wen-Shiann Wu

**Affiliations:** a Division of Cardiovascular Medicine, Chi-Mei Medical Center; b Department of Public Health of Medicine, College of Medicine, National Cheng Kung University; c Department of Green Energy and Environmental Resources; d Department of Occupational Safety and Health, Chang Jung Christian University; e Division of Cardiovascular Medicine, Chiali branch of Chi-Mei Hospital; f Departments of Neurology and Occupational Medicine, Chi-Mei Medical Center; g Department of Pharmacy, Chia-Nan University of Pharmacy and Science, Tainan, Taiwan.

**Keywords:** cardiovascular risk, hypertension, incidence, mortality, National Health Insurance Research Database, prevalence, Taiwan

## Abstract

Hypertension continues to be an important public health concern because of its associated morbidity, mortality, and economic impact on society. The aims of this study are to compare the secular changes in age-stratified hypertension prevalence, incidence, co-morbidity, and 3 years of cardiovascular outcome in Taiwan in the years 2005 and 2010.

We enrolled hypertensive individuals from the datasets of the Longitudinal Health Insurance Database (LHID) in 2005 and 2010 in Taiwan separately. We analyzed the hypertension prevalence, incidence, medication treatment, and associated morbidities. The risks of cardiovascular and cerebrovascular events and all-causes mortalities among the hypertensive individuals were evaluated in 3 years of follow-up.

There was an increased prevalence of hypertension but decreased incidence of hypertension in those over 65 from 2005 to 2010. Dyslipidemia was the highest rate of co-morbidity in 2005 and 2010. The most frequent categories of anti-hypertensive agents prescribed was 1 or 2 for both 2005 and 2010. Calcium channel blockers were the most common anti-hypertensive agents prescribed, followed by Angiotensin converting enzyme inhibitors/Angiotensin receptor blockers. After 3 years of follow-up, the risks of coronary artery disease (CAD), cerebrovascular diseases (CVD) as well as death were less in 2010 than in 2005 in Taiwan.

Our study showed that hypertension individuals had an increased prevalence, younger age, decreased incidence, increased medication treatment associated with decreased the CAD, CVD, and mortalities in 2010 compared to 2005 in Taiwan.

## 1 Introduction

As a well-known risk factor for cardiovascular morbidity and mortality,^[1]^ hypertension is a chronic disease with much world attention. It has been reported hypertension was present in 69% of patients who experienced their first heart attack, and 77% of those suffering from their first stroke.^[2–5]^ Prevalence estimates were significantly higher in the elderly (≥65 years old) compared with young adults (<65 years old).^[6]^ Previous research also reported the effective reduction of blood pressure is closely related to decreased cardiovascular disease and stroke.^[4]^ Additionally, antihypertensive therapy is directly associated with reduced blood pressure. A systematic review compiling 1479 related studies in these 40 years indicated the global prevalence of adult hypertension increased from 26.4% in 2000 to 31.1% in 2010, 28.5% in high-income countries such as the West and Asia Pacific area and 31.5% in low- and middle-income countries such as East Asia, Southeast Asia, South Asia, Oceania, and the South African region below the Sahara.^[7]^ An estimated 1.39 billion people had hypertension in 2010. From 2000 to 2010, the age-standardized prevalence of hypertension decreased by 2.6% in high-income countries but increased by 7.7% in low- and middle-income countries.^[7]^

A lot of research clearly shows the occurrence of high blood pressure increases the number of complications and even death, bringing a heavy financial burden to a country.^[8,9]^ Therefore, monitoring blood pressure changes in epidemiological data, treatment type, and the quality of care must be ongoing. Early recognition and treatment of hypertension is important to improve morbidity and mortality, especially for cardiovascular and cerebrovascular diseases (CVD). Even in hemodialysis patients, blood pressure control was independently associated with all-cause mortality and cardiovascular events.^[10]^

Generally, the treatment of hypertension can be divided into 2 parts: lifestyle modification changes and drug treatment. The former includes salt and alcohol intake restrictions, weight loss, smoking cessation, diet control, and exercise.^[11,12]^ The latter would be various recommendation treatment guidelines in different countries and times, such as WHO/ISH; Prevention, Detection, Evaluation and Treatment of High Blood Pressure by the US Joint National Committee (JNC); the British Hypertension Society (BHS); and the European Society of hypertension / European Society of Cardiology (ESH / ESC), etc.^[13]^ Taking JNC7 published in 2003 for instance, hypertensive patients in stage I (140/90 mm Hg) are recommended thiazide-type diuretics, as well as other drugs such as angiotensin-converting enzyme inhibitors (ACEIs), angiotensin receptor blockers (ARBs), beta-blockers (BBs), calcium channel blockers (CCBs), and others if complications existed. In addition, patients with different severity and treatment goals have corresponding recommendations for the number and type of medicine.^[1]^ Two-drug combinations as a first step antihypertension treatment have been emphasized in recent years. An updated JNC8 with stronger evidence and revisions for treatment targets and medication choice was released after 10 years.^[14]^

The Taiwan Society of Cardiology also published 2 versions of guidelines for tackling high blood pressure in 2010 and 2015. These were more Asian-oriented, such as more emphasis on the importance of stroke when considering the cardiovascular prognosis.^[15,16]^ However, since following the guidelines is not compulsory in actual clinical practice, many studies focused on the prescription pattern of antihypertensive agents and factors affecting these patterns.

There have been several studies focused on the medication pattern for hypertension over the last few decades in Taiwan. In general, CCBs are the class of medication with the highest use frequency. Diuretics are uncommon on the other hand; and there has been a gradual trend for the replacement of ARBs to ACEIs.^[17]^ The most commonly used medication varies according to the country. For instance, ACEIs are the most frequently prescribed medications in Canada, the United Kingdom, and the United States; while BBs are popular in Finland, Iceland and Sweden; CCBs are often prescribed in Norway and Denmark.^[18]^ The factors leading to distinct prescription pattern involve the individual-level (such as sex, age, comorbidity) and environmental-level (such as physician specialty, institution level, healthcare insurance system, country). The findings of this study explore the real public health data of hypertension, confirm the health care achievements of NIH in Taiwan, and are anticipated to provide a reference not only for caregivers but also for the policy makers of national health insurance to achieve reasonable and cost-effective drug recommendations.

## 2 Methods

To evaluate secular changes in the prevalence and incidence of hypertension and the subsequent cardiovascular or CVD and mortality, we conducted a retrospective study cohort study by analyzing 2 independent datasets of the Longitudinal Health Insurance Database (LHID) 2005 and LHID 2010.

### 2.1 Data source

The outpatient records dataset of the LHID 2005 and LHID 2010 were used in this study. Both LHID 2005 and LHID 2010 contain the entire original claim data of 1,000,000 beneficiaries randomly sampled from the corresponding year of the National Health Insurance Research Database (NHIRD). This corresponded to about 4% of all enrollees in the National Health Insurance program.^[19]^ The Health Insurance program is a nationwide healthcare system in Taiwan established in 1995, and its coverage rate exceeded 99% in 2007. There were no significant differences in the age and sex distribution between the selected sample and all enrollees.

The LHID contains information on each member, including an encrypted personal identification number, sex, date of birth, diagnostic codes using the International Classification of Diseases, Ninth Revision, Clinical Modification (ICD-9-CM), drug prescriptions, medical cost, medical care facilities, and specialty of care providers. All datasets are interlinked by the personal identification number of the patient.

### 2.2 Study population and study design

**Figure 1:**
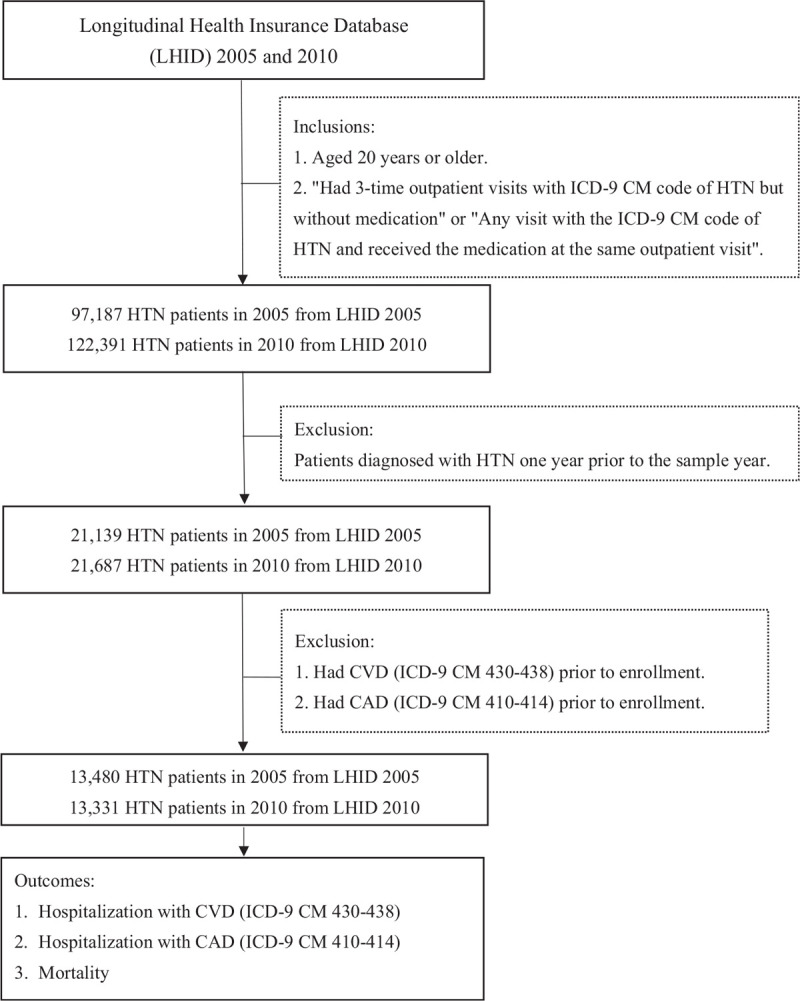
Study Diagram.

The study diagram is presented in Figure 1.

#### 2.2.1 Prevalence rate

Individuals whose records showed ICD-9-CM codes 401–405 for 3 outpatient visits, or those prescribed an antihypertensive agent at any outpatient visit were diagnosed as hypertensive from January 1^st^ to December 31st for the year 2005 from the LHID 2005 dataset and 2010 from the LHID 2010 dataset, respectively, to evaluate the prevalence of hypertension by age group. The date of diagnosis of hypertension was the earlier date of either: the third outpatient visit, or the first outpatient visit with an antihypertensive prescription.

#### 2.2.2 Incidence rate

Further, hypertensive individuals would be enrolled after excluding the hypertension history 1 year prior to the year of enrollment. The incidence of hypertension was calculated by age groups in the 2 different datasets.

#### 2.2.3 Comorbidity evaluation

To compare the history of the concomitant conditions of patients between 2005 and 2010, outpatient records were traced for up to 1 year prior to the date of hypertension diagnosis for any diagnosis containing the following ICD-9-CM codes: 250 (diabetes mellitus), 272 (hyperlipidemia), 440–459 (peripheral vascular disease), 140–208 (cancer), 580–589 (renal disease), 490–496 (lung disease), 531–534 (ulcer). Patients with records of any of the above-mentioned codes during that time were considered to have the corresponding comorbid conditions.

For evaluating CAD and CVD risk, hypertensive individuals with the ICD-9-CM code 410–429 (heart disease), 430–432 (hemorrhagic stroke), and 433–438 (ischemic stroke) prior to the individuals were excluded.

#### 2.2.4 Medication categories for hypertension treatment

Antihypertensive agents were categorized into 6 major categories, including alpha-blockers, BBs, CCBs, diuretics, ACEIs or ARBs, and others. This study examined the prescription of antihypertensive medication classes in hypertensive individuals on the date of diagnosis and the number of agent(s) prescribed. ACEIs and ARBs were treated as 1 class because of their similar treatment effects.

#### 2.2.5 CAD and CVD risk and all-causes mortality in hypertensive individuals

Hypertensive individuals were followed up for 3 years. Individuals hospitalized for coronary artery disease (CAD) (ICD-9-CM 410–414) including myocardial infarction and angina (ICD-9-CM 410–414), and CVD ((ICD-9-CM 430–438) were identified as outcome event. We also compared the events of all-cause deaths (withdrew from National Health Insurance after enrollment) for 3 years.

### 2.3 Statistical analysis

For comparing 2005 and 2010, the Chi-Squared test was used for categorical variables and *t*-test for continuous variables. Subsequently, univariate and multivariate binary logistic regression was performed for the likelihood of prescribing any anti-hypertensive agents, as well as each type of agent. The different outcomes of 3-year cumulative incidences were analyzed by Cox proportional Hazard models. The univariate factors with *P* < .05 were then entered into the multivariate model. *P* < .05 was considered statistically significant. All the data processing and statistical analysis were performed using SAS statistical software (SAS Institute Inc., Version 9.4, Cary, NC). The study was approved by the Institutional Review Board at the Chi-Mei Medical Center.

## 3 Results

### 3.1 The prevalence, incidence, and characteristics of hypertension in 2005 and 2010

**Figure 2:**
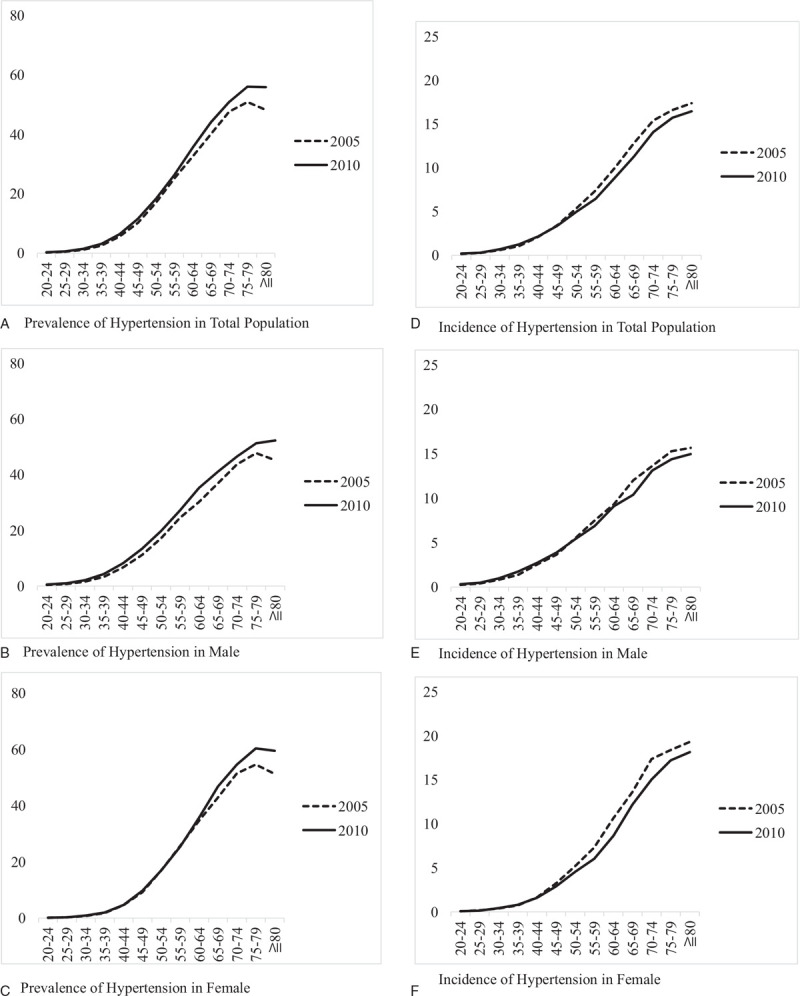
Prevalence and incidence of hypertension in 2005 and 2010 at different age group. Prevalence of hypertension in total population. Prevalence of hypertension in male. Prevalence of hypertension in female. Incidence of hypertension in total population. Incidence of hypertension in male. Incidence of hypertension in female.

There were 97,187 (13% of sample population) 122,265 (15.71% of sample population) individuals meeting the criteria of hypertension in 2005 and 2010, respectively (Fig. 1). There was increased prevalence of hypertension (Fig. 2a-c) but decreased incidence of hypertension in females and especially older age groups of 65 to 80 years old or more (Table 1, Fig. 2d-f).

**Table 1:**
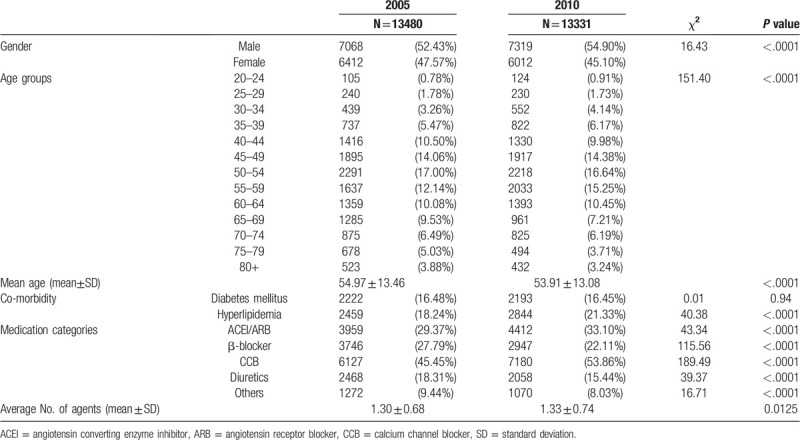
Characteristics of patients with incident hypertension, co-morbidity and medication for hypertension in two groups from 2005 to 2010 in Taiwan.

Characteristics of the hypertensive population are summarized in Table 1. The proportion of males was 54.9% in 2010, which was higher than 52.43% in 2005 (*P* < .001) (Fig. 2b), and the age distribution of both cohorts was concentrated between 45 and 64 years old (Table 1). Among comorbidity in 2005 and 2010 during follow-up, dyslipidemia showed the highest rate of comorbidity in 2005 (25.86%) and 2010 (32.93%), and the rate increased in 2010 (*P* < .001) (Table 1).

Of the types of anti-hypertensive agents prescribed (Table 1), the rankings in both cohorts are the same, which are CCBs, ACEIs/ARBs, beta-blockers, diuretics, alpha-blockers, and then others. CCBs, diuretics, and ACEIs or ARBs showed increased use while alpha-blockers, beta-blockers, and others decreased.

The most frequent number of anti-hypertensive agents prescribed was 1, 2 and none for both 2005 and 2010. For more details, more patients were not prescribed any agent (13.29%–15.95% from 2005 to 2010); fewer patients were prescribed 1 agent (44.79%–41.78% from 2005 to 2010). The trends for the proportions of patients with 2 agents were stable (29%). The mean numbers of antihypertensive drugs were about 1.3 in both the 2005 and 2010 cohorts (Table 1).

### 3.2 The 3- year-cardiovascular outcomes in 2005 and 2010

**Figure 3:**
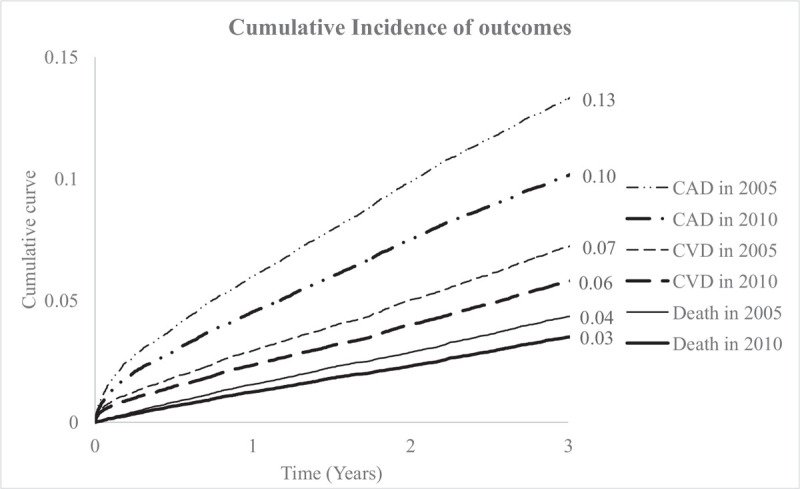
Three years cumulative incidence of coronary artery diseases, CVD cerebrovascular diseases and mortality outcomes of hypertension individuals since 2005 and 2010.

Patients were followed up for 3 years for the CAD, CVD and all-causes mortality. Cox proportional hazard models stratified by sex and adjusted for age and co-morbid condition were constructed to determine the development of cardiovascular, cerebrovascular endpoints as well as death. The Hazard Ratio (HR) revealed the cumulative incidence of CAD is 0.13 (in 2005) vs 0.10 (in 2010) (*P* < .0001), the CVD is 0.07 (in 2005) vs 0.06 (in 2010) (*P* < .0001) and the death is 0.04 (in 2005) vs 0.03 (in 2010) (*P* < .0001) (Table 1 and Fig. 3).

## 4 Discussion

To understand whether the model of antihypertension drugs is reliable for health insurance and medical source distribution, this study used Taiwan's National Health Insurance data base to analyze the hypertension prevalence, incidence, co-morbidity, drugs used, 3-year risk of vascular events, and all-cause mortality.

The study showed the overall prevalence of hypertension increased, but decreased in females and the elderly. The secular changes in the prevalence and incidence of hypertension were associated with the early recognition and treatment of hypertension and the improved treatment of cardiovascular and cerebrovascular disease attack and mortality prevention in Taiwan. In the Taiwan's National Health Insurance system, there was a relatively high awareness among health care professionals and the public as to the importance of hypertension in 2010 compared to 2005. Blood pressure is an important, modifiable, risk factor, and noteworthy for being easily measured by non-physician healthy providers. The major co-morbidity is dyslipidemia and this had increased in 2010. This was related to more obese patients and a higher cholesterol diet intake in Taiwan.^[20]^ In a prevalence study in Korea, a similar result was found.^[21]^ Hypertension had the highest overall prevalence rates.

The prescribed anti-hypertension drugs showed more popular use of ACEIs /ARBs and CCBs. This was because of the target organ protective effect of ACEIs/ARBs being more emphasized by JNC8 and Taiwan Society of Cardiology guidelines^[15,16]^ and the greater long-time potent effect of CCBs.

In our study, the mean numbers of antihypertensive drugs were about 1.3 in both 2005 and 2010. This information helps us modify the clinical practice that early combination therapy is better than monotherapy to achieve the target blood pressure.^[22,23]^ Both the 2003 American and the 2007 and 2013 European Society of Cardiology/European Society of Hypertension guidelines advised initial use of 2 antihypertensive drugs in selected hypertensive groups of patients.^[23–26]^ For the American guidelines, an initial 2-drug combination was recommended when the baseline BP was at least 20/10 mm Hg (systolic/diastolic) above the target of <140/90 mm Hg.

The 3-year-outcome revealed less cumulative incidence of CAD, CVD, and deaths in 2010 than in 2005. This was partly due to more patients being included in the National Health Insurance and greater awareness of hypertension and its complications. More people received early treatment of hypertension and more potent end organ protective drugs were prescribed.

There are some limitations to our study. First, we were unable to obtain the blood pressure (BP) measurement data of the patients in the LHID 2010 and 2005. Blood pressure control, especially high systolic blood pressure and low diastolic blood pressure, is related to the clinical cardiovascular and cerebrovascular outcome.^[27–32]^ Owing to the limitations of real-world blood pressure information, we could not offer the BP management result that may be needed after full consideration of age and sex which can significantly influence health behaviors. Second, the LHID 2010 and 2005 did not include personal risk factors for hypertension, including smoking, body mass index, exercise, salt intake, and drinking. These risk factors may influence the prevalence of hypertension, but the confounding factor may be small because of this studys large sample size which is needed to evaluate the effects of many risk factors simultaneously. Future directions of the study should include the blood pressure data of the patients because the treatment rate and blood pressure level are related to the cardiovascular events.^[9,27–32]^ The third limitation is we did not have LHID datasets earlier than 2005 or later than 2010 and these have helped in understanding these trends. This also aims our consideration for future study to collect more variables about the clinical patient data, including BMI, waist circumference, metabolic syndrome, lipids, exercise, smokers, liver steatosis, alcohol intake, lung disease¸ gout, atrial fibrillation, high-sensitivity C-reactive protein, Fasting glucose, depression, valve disorder, complete blood tests, family history of stroke, CAD, peripheral arterial disease, and stroke subtypes which may significantly affect clinical decisions.

In conclusion, in Taiwan, the hypertension incidence rates among those over 65 years old decreased in 2010 compared to 2005. CCB and ARB/ACEI were the top 2 most common categories prescribed for hypertension and more than 1 drug category used on average, suggesting more aggressive treatment as well as combination treatment as the first step in antihypertensive therapeutic intervention.^[22,25]^ Due to the convenience of the National Health Insurance System in Taiwan, more patients have access to affordable health care for hypertension and as a result the CAD, CVD, and mortalities in hypertensive individuals in Taiwan have improved.

## Author contributions

**Conceptualization:** Chia-Te Liao, Jung-Chang Shih, Tain-Junn Cheng, Wen-Shiann Wu.

**Data curation:** Chia-Te Liao, Pei-Chih Wu, Jung-Chang Shih, Tain-Junn Cheng.

**Formal analysis:** Chia-Te Liao.

**Funding acquisition:** Tain-Junn Cheng.

**Investigation:** Chia-Te Liao, Jung-Chang Shih, Tain-Junn Cheng.

**Methodology:** Chia-Te Liao, Pei-Chih Wu, Tain-Junn Cheng, Wen-Shiann Wu.

**Project administration:** Wen-Shiann Wu.

**Validation:** Wen-Shiann Wu.

**Visualization:** Wen-Shiann Wu.

**Writing – original draft:** Chia-Te Liao, Wen-Shiann Wu.

**Writing – review & editing:** Wen-Shiann Wu.
